# Estratificação do Risco de Mortalidade na Insuficiência Cardíaca. A Busca pelo Santo Graal Continua! A Análise do Sistema Nervoso Autônomo está de Volta!

**DOI:** 10.36660/abc.20230761

**Published:** 2024-02-15

**Authors:** Eduardo Arrais Rocha, Bianca Lopes Cunha, Helena Nogueira Brasil, Francisca Tatiana Moreira Pereira, Roberto da Justa Pires

**Affiliations:** 1 Faculdade de Medicina Universidade Federal do Ceará Fortaleza CE Brasil Faculdade de Medicina da Universidade Federal do Ceará, Fortaleza, CE – Brasil; 2 Centro de Arritmia do Ceará Fortaleza CE Brasil Centro de Arritmia do Ceará, Fortaleza, CE – Brasil; 3 Universidade Federal do Ceará Hospital Universitário Walter Cantídio Fortaleza CE Brasil Universidade Federal do Ceará - Hospital Universitário Walter Cantídio, Fortaleza, CE – Brasil; 4 Hospital São José de Doenças Infecciosas Fortaleza CE Brasil Hospital São José de Doenças Infecciosas, Fortaleza, CE – Brasil

**Keywords:** Insuficiência Cardíaca, Medição de Risco/métodos, Mortalidade, Sistema Nervoso Autônomo

Gomes et al.,^1^ descreveram interessante estudo evidenciando que a análise do sistema nervoso autônomo (SNA) deva participar na avaliação prognóstica dos pacientes com insuficiência cardíaca (IC). Uma nova forma de avaliação do simpático foi analisada, com a mensuração de intervalos de 10 minutos da variabilidade da frequência cardíaca (VFC), no domínio do tempo, em Holters de 24hs de pacientes internados e predominantemente com IC.

A relevância clínica do trabalho está em demonstrar que a avaliação de variáveis de curtos períodos da VFC é um dos parâmetros mais sensíveis, pois detecta breves momentos de aumento do tônus simpático, que parecem estar mais associados às descompensações da IC e ao aumento de morte súbita cardíaca (MSC), adicionando um parâmetro (menor rMSSD), a um modelo preditor de risco que inclui a idade > 69 anos e a fração de ejeção (FE) <57%, com boa acurácia.

A principal vantagem do registro de VFC de curta duração é a fácil aplicação e aquisição dos intervalos RR em condições controladas como repouso ou teste de esforço. Na literatura, encontramos estudos que utilizaram registros de curta duração com tempos que variam de 1 minuto a 60 minutos.^2,3^

Shah et al.,^4^ evidenciaram associação entre a redução dos índices da VFC e o aumento da incidência de IC em pacientes hipertensos em seguimento de 7,6 anos. LaRovere et al.,^5^ demonstraram uma associação da redução da VFC com aumento de morte súbita em pacientes com IC em seguimento de 3 anos. Ambos os estudos usaram variáveis de curtos períodos da VFC.

A predição de risco de mortalidade na IC é complexa. Alguns estudos sobre os marcadores preditores de mortalidade foram publicados.^6^ A maioria dos modelos concentrou-se na predição de hospitalização por IC e não em mortalidade, tendo apresentado desempenho apenas mediano e usado parâmetros de mais difícil análise.^7^

As limitações do estudo são bem descritas pelos autores e merecem destaques,^1^ como: o pequeno número de casos, a heterogeneidade da população, a inclusão de pacientes com síncopes e ser um estudo retrospectivo com diversos vieses potenciais. Certamente essas limitações demonstram a necessidade de novos estudos com maior casuística, prospectivos, que possam ou não confirmar esses achados. A necessidade de validação interna e externa dos modelos também são fundamentais.

Diversas variáveis têm sido testadas nas últimas décadas na tentativa de se identificar o modelo mais acurado ou a variável mais forte na estratificação do risco de mortalidade em pacientes com IC.^8^ As diferenças entre as características das diversas etiologias de miocardiopatia têm sido uma das principais limitações. A FE passou a ser o parâmetro mais próximo do “Santo Graal”, mas quando se dicotomiza entre etiologias isquêmicas e não isquêmicas (CNI), ela perde poder estatístico na CNI. Certamente como disse o Dr. Alfred Buxton, autor do estudo MUSTT: “a FE <30 % não é tudo que precisamos para decidir sobre o implante de um desfibrilador cardíaco automático (CDI),. Na mesma controvérsia, o Dr. Arthur Moss, autor dos estudos MADIT disse: “a FE < 30 % é tudo o que precisamos para decidir sobre o implante de um CDI”.^9,10^

Em algumas análises realizadas,^11,12^ os parâmetros de estudo do SNA mostraram-se relevantes e com significância estatística para prever o risco de mortalidade total e de MSC. Entretanto, quando são realizadas análises mais robustas em modelos de regressão multivariados, seus parâmetros perdem poder estatístico, sendo excluídos como variáveis independentes. Isso não significa que não sejam importantes e sim que seus parâmetros podem já estar sendo demonstrados indiretamente em outras variáveis. Por exemplo, pacientes com FE muito reduzidas, usualmente apresentam aumento no tônus simpático. Entretanto nas análises conjuntas dos 2 parâmetros, os testes de VFC não mantêm poder estatístico como preditores independentes.

Pequenos períodos de observação da VFC avaliam predominantemente a atividade do SNA parassimpático, e, portanto, não avaliam adequadamente a atividade simpática, questionando-se por esse motivo a validade da análise de variáveis no domínio do tempo em registros de curta duração e gerando muitas controvérsias na Literatura. Por outro lado, alguns estudos mostraram que rMSSD e o pNN50, também variáveis do domínio do tempo que representam essa atividade parassimpática, podem ser analisadas em curtos registros, pois sua análise é realizada a partir de intervalos RR adjacentes.^13^

Mais estudos precisam ser realizados para investigar o real valor prognóstico da VFC em paciente com IC, empregando: (1) diferentes métodos para analisar os dados de VFC, (2) diferentes períodos de tempo após a descompensação da IC para avaliar a VFC basal, (3) diferentes períodos de acompanhamento (de meses a anos) e (4) diferentes desfechos (por exemplo, eventos arrítmicos, MSC e mortalidade cardíaca e mortalidade total), a fim de estabelecer quais os reais pontos de cortes da VFC que vão associar-se com pior prognóstico.^14^

Dessa forma o estudo de Gomes et al.,^1^mostrou-se oportuno por resgatar o debate de um tema muito importante e por sugerir uma nova estratégia de avaliação do SNA em pacientes internados com IC ou síncopes. Aguardemos a confirmação dos dados apresentados para efetivação desses parâmetros na prática clínica.
