# Supplementary material for: Estratificação do Risco de Mortalidade na Insuficiência Cardíaca. A Busca pelo Santo Graal Continua! A Análise do Sistema Nervoso Autônomo está de Volta!
Source: Arq Bras Cardiol. 2024 Feb 15;120(11):e20230761. [Article in Portuguese] doi: 10.36660/abc.20230761 (PMC11098587; doi:10.36660/abc.20230761)
Supplement: Translation

# Translation: Mortality Risk Stratification in Heart Failure. The Search for the Holy Grail Continues! Autonomic Nervous System Analysis is Back!

**Authors:** Eduardo Arrais Rocha, Bianca Lopes Cunha, Helena Nogueira Brasil, Francisca Tatiana Moreira Pereira, Roberto da Justa Pires

**Affiliations:** 1 Brazil Faculdade de Medicina da Universidade Federal do Ceará, Fortaleza, CE – Brazil; 2 Brazil Centro de Arritmia do Ceará, Fortaleza, CE – Brazil; 3 Brazil Universidade Federal do Ceará - Hospital Universitário Walter Cantídio, Fortaleza, CE – Brazil; 4 Brazil Hospital São José de Doenças Infecciosas, Fortaleza, CE – Brazil

**Keywords:** Heart Falure, Risk Assessment/methods, Mortality, Autonomic Nervous System

Gomes et al.^1^ described an interesting study showing that the analysis of the autonomic nervous system (ANS) should participate in the prognostic assessment of patients with heart failure (HF). A new form of sympathetic assessment was analyzed, measuring 10-minute intervals of heart rate variability (HRV) in the time domain on 24-hour Holters of hospitalized patients, predominantly with HF.

The clinical relevance of the work lies in revealing that the evaluation of short-term HRV variables is one of the main parameters, as it detect brief moments of increased sympathetic tone, that seems to be more associated with HF decompensation and an increase in sudden cardiac death (SCD), adding a parameter (smallest rMSSD) to a risk prediction model that includes age > 69 years and ejection fraction (EF) <57%, with good accuracy.

The main advantage of short-term HRV recordings is the easy application and acquisition of RR intervals under controlled conditions such as rest or exercise testing. In the literature, we found studies that used short-term recordings with times ranging from 1 minute to 60 minutes.^2,3^

Shah et al.^4^ showed an association between the reduction in HRV indices and the increased incidence of HF in hypertensive patients over a 7.6-year follow-up. LaRovere et al.^5^ demonstrated an association between reduced HRV and increased sudden death in patients with HF over a 3-year follow-up. Both studies used short-term HRV variables.

Predicting mortality risk in HF is complex. Some studies on mortality predictor markers have been published.^6^ Most models focused on predicting hospitalization for HF and not mortality, having presented only average performance and using parameters that are more difficult to analyze.^7^

The limitations of the study are well described by the authors and deserve highlights,^1^ such as the small number of cases, the heterogeneity of the population, the inclusion of patients with syncope, and is a retrospective study with several potential biases. These limitations certainly demonstrate the need for new studies with larger, prospective samples, which may or may not confirm these findings. The need for internal and external validation of the models is also fundamental.

Several variables have been tested in recent decades in an attempt to identify the most accurate model or the strongest variable in stratifying the risk of mortality in patients with HF.^8^ The differences between the characteristics of the different etiologies of cardiomyopathy have been one of the main limitations. EF has become the closest parameter to the “Holy Grail”, but when it is dichotomized between ischemic and Non-Ischemic cardiomyopathy (NICMP), it loses statistical power in NICMP. Certainly, as Dr. Alfred Buxton, author of the MUSTT study, said: “EF <30% is not all we need to decide about implanting an automatic cardiac defibrillator (ICD). In the same controversy, Dr. Arthur Moss, author of the MADIT studies, said: “EF < 30% is all we need to decide about implanting an ICD”.^9,10^

In some analyses carried out,^11,12^ the ANS study parameters proved to be relevant and statistically significant for predicting the risk of total mortality and SCD. However, when more robust analyses are performed in multivariate regression models, their parameters lose statistical power and are excluded as independent variables. This does not mean that they are not important, but rather that their parameters may already be demonstrated indirectly in other variables. For example, patients with very reduced EF usually present an increase in sympathetic tone. However, in joint analyses of the 2 parameters, HRV tests do not maintain statistical power as independent predictors.

Short periods of HRV observation predominantly evaluate the activity of the parasympathetic ANS and, therefore, do not adequately evaluate sympathetic activity, thus questioning the validity of analyzing variables in the time domain in short-term recordings and generating many controversies in the literature. On the other hand, some studies have shown that rMSSD and pNN50, also time domain variables that represent this parasympathetic activity, can be analyzed in short recordings, as their analysis is performed from adjacent RR intervals.^13^

More studies need to be carried out to investigate the real prognostic value of HRV in patients with HF, employing (1) different methods to analyze HRV data, (2) different periods after HF decompensation to evaluate baseline HRV, (3) different follow-up periods (from months to years) and (4) different outcomes (for example, arrhythmic events, SCD and cardiac mortality and total mortality), in order to establish which real HRV cutoff points will be associated with a worse prognosis.^14^

In this way, the study by Gomes et al.,^1^proved to be timely for reviving the debate on a very important topic and for suggesting a new strategy for assessing the ANS in patients hospitalized with HF or syncope. We await confirmation of the data presented to implement these parameters in clinical practice.
